# Advances and Challenges in the Development of New and Novel Treatment Strategies for Eosinophilic Esophagitis (EoE)

**DOI:** 10.3390/ph18091359

**Published:** 2025-09-11

**Authors:** Ivna Olic, Piero Marin Zivkovic, Ivan Zaja, Nikola Pavlovic, Marko Kumric, Josko Bozic

**Affiliations:** 1 Department of Gastroenterology, University Hospital of Split, 21000 Split, Croatia; ivna1211@gmail.com (I.O.); piero.zivkovic@gmail.com (P.M.Z.); ivan.zaja@yahoo.com (I.Z.); 2 Department of Internal Medicine, University of Split School of Medicine, 21000 Split, Croatia; 3 Department of Pathophysiology, University of Split School of Medicine, 21000 Split, Croatia; nikola.pavlovic@mefst.hr (N.P.); marko.kumric@mefst.hr (M.K.); 4 Laboratory for Cardiometabolic Research, University of Split School of Medicine, 21000 Split, Croatia

**Keywords:** eosinophilic esophagitis, immune-mediated disease, biologic therapy, pharmacologic treatment

## Abstract

Eosinophilic esophagitis (EoE) is a long-term, immune-driven condition of the esophagus, which can lead to severe fibrostenosis of the esophagus, and the aim is to control clinical, endoscopic, and histopathologic disorder activity. Currently, treatment options include the use of proton pump inhibitors, topical steroids, and dietary elimination as basic treatments; however, the introduction of dupilumab has provided an additional therapeutic approach. Numerous biologic agents target specific immune pathways, which are promising pharmacologic options in managing this progressive disease. The final goal is to treat the target, with complete resolution as the final objective. To accomplish this, however, effective agents capable of modifying the disease process are required. In this review, we aimed to provide an overall review of EoE therapeutics options, as well as the benefits and safety of new treatment strategies for EoE.

## 1. Introduction

Eosinophilic esophagitis (EoE) is a long-standing, progressive condition of the esophagus driven by immune mechanisms. It is marked by eosinophil-rich inflammation of the esophageal mucosa and typically presents with symptoms of esophageal dysfunction, including difficulty swallowing and episodes of food impaction [1]. A recent systematic review and meta-analysis demonstrated that the global pooled incidence is 5.31 occurrences per 100,000 person-years, with a prevalence of 40.04 occurrences per 100,000 individuals, underscoring its growing impact on public health [2]. Prevalence is increased in males, in subjects with a history of atopic diseases, and those with a family history of EoE. Also, it is higher in the European ethnic population than in Asians and African Americans. Hence, EoE is now recognized as an important contributor to gastrointestinal morbidity in both pediatric and adult populations [3,4]. It has become one of the most common causes of dysphagia [5]. Establishing a diagnosis relies on biopsy findings of at least 15 eosinophils per high-power field, after the elimination of alternative triggers of esophageal eosinophilia such as GERD or infection, drug reaction, connective tissue disease, and eosinophilic gastroenteritis [6].

Although the precise etiology of EoE remains unknown, its strong association with allergic diseases is well established. Several lines of evidence support this link, including the frequent coexistence of other atopic features, including peripheral eosinophilia, increased total IgE (tIgE), sensitization to specific IgE (sIgE) against inhalant allergens [more frequently observed in adults] and food allergens [more common in children], seasonal symptom fluctuations related to pollen exposure, improvement with elimination diets, and relief of symptoms after introducing steroid treatment. In recent years, the increasing recognition of EoE among patients undergoing oral immunotherapy has further reinforced the suspected allergic basis of the disease [7].

The presence of eosinophils in the esophageal tissue is confirmed when no alternative origins of eosinophilia can be identified [8]. The condition is driven by a T helper 2 (Th2)-dominated immune response, with food antigens identified as the primary activators [9]. The clinical presentations of EoE vary depending on the subject’s age, disease activity, progression, and stage of inflammation or fibrosis [10,11].

There are three distinct clinical phenotypes depending on the degree of inflammation and extracellular matrix deposition, namely inflammatory, fibrostenotic, or a combination of both [12]. Despite this classification, there is currently no universally accepted definition of fibrostenotic EoE, which hinders both clinical management and research efforts [13]. This lack of consensus complicates the development of targeted therapies aimed at addressing fibrostenosis. Furthermore, significant variability persists in the diagnostic approaches used to detect fibrostenotic changes in EoE patients [14,15].

The essential purpose of EoE treatment is to facilitate symptoms such as dysphagia, heartburn, nausea, and chest pain. Despite substantial advances in recent years, many subjects with EoE continue to experience suboptimal clinical outcomes or encounter considerable therapy-related side effects, highlighting the ongoing need for improved therapeutic strategies and long-term disease management.

Clinical symptoms in EoE may not reliably reflect the underlying disease activity, as pathological changes can continue to progress within the deeper layers of the esophageal wall even when symptoms appear well controlled [16]. Symptoms often emerge as late indicators of disease advancement. Therefore, only a combination of endoscopic evaluation and histological assessment through biopsy can accurately detect subclinical progression and identify patients who remain histologically active despite ongoing treatment [17].

The standard therapeutic framework is often summarized as the three Ds, which are “Drugs”, “Diet,” and “Dilation” [18]. There will be no discussion about endoscopic treatment in this paper.

Dupilumab [19] and budesonide oral suspension (BOS) [20] are currently the only two drugs that have received approval from the U.S. Food and Drug Administration (FDA) for the treatment of EoE. This limited therapeutic arsenal highlights the urgent need to advance research into novel treatment strategies and expand the current scope of EoE management.

This article aims to provide a comprehensive overview of these transformative developments, with a particular focus on recent advances in the pharmacological management of EoE.

## 2. Literature Search Strategy

Matching studies published from the past decade were identified via a PubMed Central database/Medline (accessed on 17 July 2025) search using the following keywords or combinations of keywords: eosinophilic esophagitis, treatment, monoclonal antibodies, biologics, small molecules, proteomics, microbiome, and personalized medicine. Clinical trials were retrieved from the primary clinical trials databases (www.clinicaltrials.gov and www.clinicaltrialsregister.eu) (accessed on 17 July 2025). Special attention was given to clinical trials, as well as cohort, case–control, and cross-sectional studies and guidelines addressing EoE management in adults. A systematic approach, such as reviews and meta-analyses, was excluded. We considered studies that addressed any nonendoscopic or noninvasive aspect of EoE management. Pediatric data and non-English publications were not implemented. Exclusion criteria also included case reports and conference abstracts. Data were collected and selected according to their relevance to the subject matter. When multiple studies reported overlapping patient cohorts, the most recent or comprehensive publication was selected.

Because this is a narrative review, formal quality assessment and risk-of-bias analysis were not performed. The focus was on synthesizing key findings and highlighting emerging concepts rather than quantitatively pooling data. Limitations of this approach include potential selection bias and the inability to formally compare treatment efficacy across studies.

After the initial screening of titles and abstracts on PubMed, a comprehensive search was conducted across additional electronic databases, including MEDLINE, Web of Science, Scopus, and Google Scholar, to identify further relevant literature.

## 3. Current Therapeutic Approaches

Treatment modalities for EoE, including dietary modifications and pharmaceutical interventions (esophageal dilation not discussed here), tend to attain clinical, histological, and endoscopic resolution; preclude disease burden related to tissue remodeling and dietary insufficiencies; alleviate the symptoms of esophageal dysfunction; and ensure an acceptable health-related quality of life.

The multidisciplinary Italian EoExpert Panel emphasizes that continuous, long-term treatment strategies for EoE are not routinely implemented in standard clinical practice. This gap is largely attributed to concerns about the potential long-term adverse effects of conventional therapies, as well as insufficient awareness of the disease’s progressive nature. However, optimal management of EoE requires a proactive, sustained therapeutic approach rather than intermittent treatment. Such continuous care is essential not only for achieving durable symptom control but also for maintaining histological remission and minimizing the risk of fibrotic complications such as esophageal stenosis [21].

The 2020 guidelines issued by the American Gastroenterological Association (AGA) in collaboration with the Joint Task Force on Allergy and Immunology Practice Parameters (JTF) outlined key recommendations for the diagnosis and management of EoE [22]. For subjects with symptomatic EoE, proton pump inhibitors [PPIs] are recommended over no treatment. The British Guidelines propose that PPIs are beneficial in achieving both clinical and histological remission and need to be applied twice daily for a minimum of 8–12 weeks before evaluating histological outcome [11]. According to the guidelines, topical glucocorticosteroids are the recommended therapeutic option over no treatment. For subjects who achieve remission after short-term topical steroid use, continuing treatment may be advisable due to a reasonably low side effect profile [22]. Dietary interventions may have different options. An elemental formula diet can be considered, although adherence can be challenging. Alternatively, food elimination diets—ranging from single-food to six-food elimination—have demonstrated efficacy but can likewise be difficult to maintain [22].

### 3.1. Dietary Modifications

Dietary intervention is the cornerstone of EoE management, with the primary goal of removing offending foods. Dietary treatment for EoE can be classified into three primary approaches, namely an empiric food elimination diet, an allergy testing-based elimination diet, and an elemental diet. Nonetheless, identifying and eliminating individual allergens often requires considerable effort and time [23].

Empirical food elimination diets (FEDs) exclude the six most common allergenic foods (dairy, eggs, soy, wheat, fish/shellfish, nuts/peanuts) from the diet. The effectiveness of such a strict regimen has been rated at about 72%. A recent meta-analysis including 1762 pediatric and adult patients with EoE compared different dietary strategies, namely the six-food, four-food (dairy, wheat, eggs, and soy), and single-food (dairy) elimination diets. Proportions of patients achieving histological and clinical remission were 61.3% and 92.8% with the six-food elimination diet, 49.4% and 74.1% with the four-food elimination diet, and 51.4% and 87.1% with the single-food elimination diet, respectively [24]. The main drawback of this diet is its strict dietary restrictions and the frequent need for endoscopies following the reintroduction of particular meals, since each time a food group is reintroduced, the histologic response should be reevaluated endoscopically [25]. Thus, a one-food elimination diet (OFED) has gained popularity [26].

The allergy testing-based elimination diet is based on the results of allergy tests (the patch test, prick test, or serum IgE-mediated food allergy tests). Regarding the two meta-analyses, histologic response rates vary between 45.7 and 50.8% [24,27]. Because of its implementation challenges and relatively low rates of histologic remission, testing-based dietary therapy is not widely adopted in clinical practice.

Elemental diets [EDs] are nutritionally complete or are supplementary formulations composed of easily absorbable components. They are specifically designed to meet daily nutritional requirements, including essential vitamins, macro- and trace minerals, fats, carbohydrates, and free amino acids while minimizing antigenic exposure [28]. In the clinical guidelines by the AGA and the JFT, EDs have been reported to achieve the highest response rates among therapy options and have received a conditional recommendation for use in EoE, but they can lead to de novo sensitization and produce acute IgE-mediated allergic reactions [22].

Although exclusive elemental diets are highly effective in treating EoE, unfortunately, their use is limited by poor compliance and, therefore, they are generally designated for individuals who are refractory to other therapeutic approaches [12].

### 3.2. Proton Pump Inhibitors

Proton pump inhibitors [PPIs] are regarded as a conventional first-line therapy for EoE, demonstrating effectiveness comparable to that of topical corticosteroids and dietary regimens [29]. However, only about half of patients with EoE achieve histologic remission with high-dose PPI therapy [30]. PPIs are convenient due to their relative simplicity and wide availability, as well as their relatively low cost [31]. Considerably, subjects with inflammatory phenotypes exhibited higher response rates than did those with the fibrostenotic form of EoE [32].

### 3.3. Corticosteroids

Topical corticosteroids are generally well tolerated with a favorable safety profile, with the most common adverse event being esophageal candidiasis [33]. Esophageal candidiasis is strongly and independently associated with treatment response to topical corticosteroids [tCS] in EoE. Individuals presenting with esophageal candida are over six times more likely to achieve histologic remission, even after controlling for potential confounders. The presence of candidiasis may serve not only as an indicator of local tCS effects and esophageal drug exposure but also possibly as a surrogate marker for drug adherence. This hypothesis warrants validation through future prospective studies [34].

The typical topical steroids given for EoE include fluticasone propionate and budesonide. Histologic remission, defined by a reduction in eosinophil count, was achieved in 71% of patients treated with budesonide and 64% of those treated with fluticasone [35].

Current advancements cover the advancement of new products, for example, orally disintegrating tablets and premixed suspensions, targeted to improve subject adherence and ease of use [36]. BOS (Eohilia^®^), administered at a dose of 2.0 mg twice daily, is an FDA-approved topical corticosteroid for the treatment of eosinophilic esophagitis in pediatric and adult patients aged 11 years and older, with approval limited to a 12-week treatment course. Currently, BOS is not authorized for use beyond this duration, and future investigation is necessary to establish its durable therapeutic effect and safety profile [37].

Budesonide orodispersible tablets (BOTs) have demonstrated safety and efficacy in phase III, double-blind clinical trials evaluating both induction and 48-week maintenance therapy for EoE. Recently, long-term effectiveness and safety were further assessed in a 96-week open-label extension (OLE) study, which showed that clinical, histologic, and endoscopic remission were sustained in the majority of subjects for up to three years. Additionally, prolonged BOT treatment was generally well tolerated [38,39]. Regulatory authorities have approved Jorveza^®^ for EoE management in adults in Europe, Canada, and Australia but not in the USA. BOTs were considered to have lower rates of histological remission in real-world practice than observed in clinical trials, mainly due to incorrect technique and cessation due to adverse events [40].

A post hoc analysis of the SHP621-301 study revealed that patients with EoE who achieved a clinicopathologic response after an initial 12-week course of BOS 2.0 mg twice daily experienced a lower rate of relapse when treatment was continued for an additional 36 weeks compared to those who underwent randomized treatment withdrawal. Using a more clinically relevant relapse definition, continued BOS therapy was associated with improved maintenance of remission. Conversely, treatment discontinuation was linked to the recurrence of both histologic activity and dysphagia symptoms in some patients during the follow-up period [41].

These advancements are anticipated to broaden the available treatment options for patients with EoE.

European guidelines recommend avoiding off-label corticosteroids [10] despite some significant clinical and histological improvement following off-label corticosteroid treatment [42]. Notably, systemic steroids are not indicated as a first-line therapy for inducing remission in EoE [12].

### 3.4. Anti IL-4/IL-13 Agent

In 2022, an IgG4 monoclonal antibody, dupilumab, targeting the IL-4 receptor alpha and reducing IL-4 and IL-13 signaling, was approved by the US FDA as the first therapy specifically for EoE [43].

While IL-4 can be regarded as a potent amplifier of the innate type 2 immune response, IL-13 is considered the central effector cytokine of the adaptive type 2 immune system [44].

**Figure 1:**
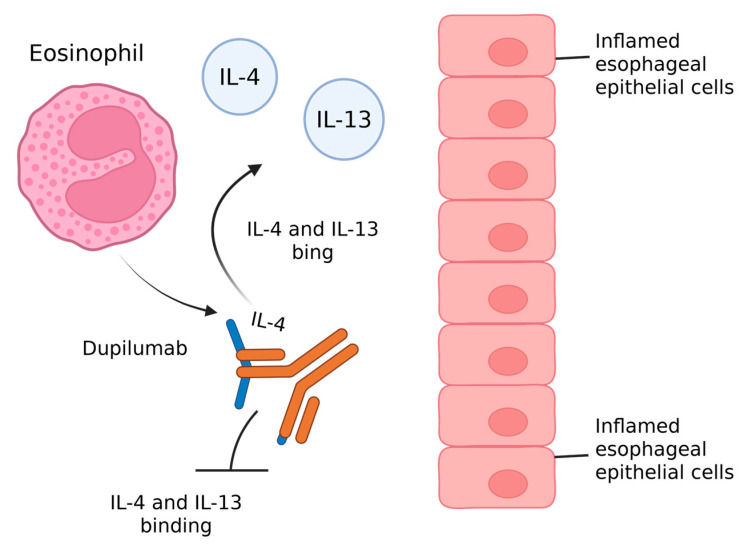
Mechanism of action of the first approved biologic therapy for EoE.

Dupilumab has demonstrated particular efficacy in patients with PPI-refractory EoE or in those who are unable to tolerate topical corticosteroids or elimination diets. Moreover, in individuals with comorbid inflammatory conditions such as asthma or atopic dermatitis, dupilumab provides the added advantage of simultaneously treating both EoE and the associated atopic disorders [45] (Figure 1).

Patients should be monitored for hypersensitivity reactions, which may include elevated blood pressure and heart rate, as well as rare ocular side effects that can occur following injection [46].

Bowyer et al.’s real-world FAERS analysis (Q1 2022–Q4 2023) indicates that dupilumab has a consistent and feasible safety profile for EoE, according to phase III trials. Considerably, older adult patients may have increased odds of serious adverse events. Clinicians should continue routine injection site monitoring and remain alert to age-related risk [47].

Biological therapy with dupilumab is currently the most expensive among the available therapies for EoE [48].

For patients who have a strong preference for avoiding topical steroids, such as those with diabetes, those experiencing candidiasis, individuals receiving multiple steroid treatments for type 2 comorbidities (diseases with type 2 inflammatory response driven mainly by T helper 2 cells, such as atopic dermatitis, asthma, and allergic rhinitis), or when there is a desire to minimize cumulative steroid exposure or for those facing significant dietary restrictions due to various food triggers, initiating therapy with dupilumab may be a viable alternative [49,50].

In dupilumab phase 3 (LIBERTY-TREET trial), subjects with an impassable esophageal stricture representing a fibrostenotic form of EoE that could not be passed with a standard upper endoscope were excluded. So, there is an urgent need to evaluate the effectiveness of biological treatment in patients who require esophageal dilation [51] (Table 1).

### 3.5. Anti IL-13 Agents

Cendakimab (RPC4046, CC-93538), a humanized IgG1k monoclonal antibody targeting IL-13, leads to reductions in histological inflammation and endoscopic findings in EoE subjects by inhibiting the interaction of IL-13 with the matching IL-13Rα1 and IL-13Rα2 receptors, demonstrating potential efficacy in a multicenter trial (HEROES) of adult subjects with active EoE. Weekly subcutaneous injections of 180 mg or 360 mg substantially ameliorated histopathologic and endoscopic results at week 16 compared to a placebo [52]. Though crucial symptoms, such as dysphagia, did not improve considerably, the high-dosage population exhibited benefits in the overall rating of disease severity. A further possible anti-fibrotic/anti-stenotic drug might be the anti-IL-13 agent cendakimab, which has already elucidated promising results in a phase 2 trial [53], and it is presently being studied in phase 3 but unfortunately also excludes subjects with severe strictures [53].

Dectrekumab (QAX576), another anti–IL-13 monoclonal antibody, was evaluated in a proof-of-concept trial for EoE. Although treatment led to a reduction in esophageal eosinophil counts compared with a placebo, the primary endpoint of histologic response was not achieved. Nevertheless, favorable changes were observed in the expression of key inflammatory mediators implicated in EoE pathogenesis [54].

### 3.6. Anti IL-5 Agents

IL-5 is a key type 2 cytokine with selective activity on eosinophils. It drives their maturation in the bone marrow, enhances the responsiveness of circulating cells, and prolongs survival once they infiltrate tissues. The IL-5 receptor consists of a specific α subunit (IL-5Rα) paired with the standard βc chain also used by IL-3 and GM-CSF receptors. Upon ligand binding, downstream signaling cascades are triggered, including JAK/STAT5, PI3K/AKT, and Ras/ERK, which collectively promote eosinophil persistence, migration, and effector functions. Therapeutic neutralization of IL-5 interrupts these pathways and results in substantial reductions in blood and tissue eosinophilia [55].

Mepolizumab and reslizumab, humanized IgG1k monoclonal antibodies aiming to bind peripheral IL-5, provided histopathological remission in EoE but had limited clinical effectiveness in both adult and pediatric cohorts [56,57].

Drug monitoring of mepolizumab therapy includes an assessment of absolute eosinophil counts in patients with hypereosinophilic syndrome, as well as vigilance for potential adverse effects such as gastrointestinal symptoms, injection site reactions, flu-like manifestations, and fatigue [58].

Benralizumab, focusing on IL-5-R, likewise induces antibody-dependent cell-mediated cytotoxicity with encouraging results but no direct connection between histopathological and clinical benefits in trials according to the MESSINA study (NCT04543409) [59]. It is currently used as an add-on therapy for subjects 12 years and older with severe eosinophilic asthma. Subjects need to be observed for anaphylaxis/hypersensitivity reactions during and after administration, as well as any signs of infection [60]. In the recent cohort, treatment with benralizumab did not succeed in resolving inflammation or epithelial dysfunction, indicating that monotherapy targeting the interleukin-5 receptor alpha is insufficient for effective disease control in EoE [61].

### 3.7. Anti-Siglec, Anti-S1P, Anti-c-KIT

Siglec-8, a sialic acid-binding immunoglobulin-like lectin selectively expressed on mature human eosinophils and mast cells, represents a strategic therapeutic target. The engagement of Siglec-8 induces the apoptosis of IL-5/IL-33, primed eosinophils through a ROS, and a mitochondria-dependent, caspase-mediated pathway, whereas in mast cells, ligation suppresses FcεRI-dependent signaling by interfering with proximal kinase cascades, thereby reducing degranulation and mediator release [62]. Lirentelimab, a humanized Ig G1 monoclonal antibody targeting Siglec-8 on the surface of mature eosinophils and mast cells, offers a dual-effector approach that directly modulates key cellular drivers of EoE pathogenesis. It was promising for managing EoE and other eosinophilic disorders. However, in the adult and adolescent cohort of a randomized, double-blind, placebo-controlled phase 2/3 study, lirentelimab did prove significant histopathologic remission but without substantial relief of symptoms [63].

Currently, a second phase of the “EvolvE” study, a randomized, double-blind, placebo-controlled trial to assess the safety and benefits of barzolvolimab, an anti c-KIT monoclonal antibody (CDX-0159) in an adult population with EoE [64], is being conducted. However, this study of barzolvolimab also excluded patients with esophageal strictures that cannot be easily passed with a standard adult upper endoscope (9–10 mm) or manifests as a stricture necessitating dilation.

Etrasimod is an orally administered, once daily, selective modulator of the sphingosine-1-phosphate (S1P) receptor, being developed for use in immune-mediated inflammatory conditions. It decreases the number of circulating lymphocytes, which helps reduce inflammation and tissue damage [65]. In a placebo-controlled, double-blind study, treatment with etrasimod resulted in durable histological and endoscopic remission over 52 weeks, along with a reduction in symptoms in affected individuals not requiring dilation, and it was well tolerated. These results represent the first demonstration that interfering with the S1P pathway can attenuate condition severity in EoE, highlighting S1P receptor modulation as a potential therapeutic avenue [66].

## 4. Drugs Acting on the Thymic Stromal Lymphopoietin (TSLP)

Tezepelumab is a human IgG2 monoclonal antibody that inhibits TLSP interaction with the matching receptor by attaching to circulating TLSP. It is presently authorized for the management of asthma [67]. TSLP interferes with a receptor formed by IL-7R and TSLP-R chains and influences various immune cell types. Current investigations have shown greater TSLP receptor sensitivity in esophageal-derived memory CD4+ T cells from EoE subjects, proposing a role in the pathophysiology of the disorder [68]. The US FDA has granted orphan drug designation to tezepelumab for the management of EoE, and ongoing clinical trials are investigating its efficacy and safety in both adults and adolescents [69,70].

EoE is a type 2 (T2) inflammatory condition considered part of the “atopic march,” alongside bronchial asthma, allergic rhinitis, atopic dermatitis, and food allergies, due to shared epidemiological, genetic, and pathophysiological features. Despite frequently co-occurring within the same individual, these atopic disorders are typically managed as separate conditions by different specialists, often leading to polypharmacy and prolonged corticosteroid use. In response, a range of biologic therapies has been developed to target common T2-mediated pathways, including those involving IL-5, IL-4/IL-13, IgE, and TSLP [71]. For all these reasons, various biological drugs targeting shared T2-related pathways (including IL-5, IL-4/IL-13, IgE, TSLP) have been developed. The link between EoE and other atopic diseases was hypothesized almost 20 years ago, when EoE was described as the “asthma of the esophagus” [72].

The majority of current biologics that are under investigation for efficacy in EoE are already approved for the treatment of allergic asthma [57,66,73,74,75,76,77,78,79,80] (Table 2).

Among the ten monoclonal antibodies evaluated (including mepolizumab and reslizumab, omalizumab, lirentelimab, infliximab, dectrekumab and cendakimab, benralizumab, vedolizumab, and natalizumab), mepolizumab demonstrated the most significant therapeutic potential, receiving a moderate recommendation based on Level 2 evidence [81]. In contrast, agents such as omalizumab, dectrekumab, and reslizumab showed limited clinical benefit in the context of eosinophilic esophagitis. Safety analyses from the FDA Adverse Event Reporting System [FAERS] indicated notable adverse effects associated with some of these biologics: mepolizumab and reslizumab were linked to serious events including asthma exacerbations, pneumonia, and adrenal insufficiency, while omalizumab was associated with reports of chronic obstructive pulmonary disease and gastroenteritis. The safety profile of dectrekumab remains inconclusive due to insufficient data [81].

Interestingly, various anti-allergic agents, including leukotriene and prostaglandin receptor antagonists, have been investigated as potential treatments for EoE. However, these therapies failed to demonstrate clinically meaningful improvements in either histological inflammation or EoE-related symptomatology [82]. Montelukast, a leukotriene D4 receptor antagonist, has been evaluated for the management of EoE. While open-label studies reported symptomatic improvement, particularly with high doses in adults and standard doses in children, no corresponding histologic remission was observed [83]. Furthermore, a randomized controlled trial conducted by Alexander and colleagues demonstrated that montelukast at a dose of 20 mg per day was not superior to a placebo in maintaining remission achieved through swallowed topical corticosteroids [84].

Calcineurin inhibitors such as tacrolimus possess strong immunomodulatory effects by inhibiting T-cell activation and cytokine production. Based on the notable accumulation of tacrolimus observed in an ex vivo porcine model of EoE using injury-induced esophageal mucosa, this study supports the potential of tacrolimus as a locally targeted therapeutic option for EoE management [85]. The role of immunosuppressive therapy in EoE remains experimental. Future studies are warranted to evaluate the feasibility of localized, low-systemic-bioavailability formulations, such as topical tacrolimus, as a targeted approach that may offer benefit in steroid-refractory or biologic-ineligible patients.

Vedolizumab is a monoclonal antibody approved by the FDA, EMA, and PMDA for treating moderate-to-severe ulcerative colitis [86]. It functions by blocking the integrin α4β7, thereby inhibiting its interaction with mucosal vascular addressin cell adhesion molecule 1 (MAdCAM-1), a key mechanism in lymphocyte trafficking. Although not approved for EoE, two case reports have documented its off-label use in this context. In one case, a 43-year-old male with both EoE and coexisting inflammatory bowel disease experienced marked improvement in dysphagia following vedolizumab treatment [87]. In another case, a 42-year-old female attained complete histologic remission after half a year of therapy, with endoscopic biopsies showing no detectable eosinophils [88].

Although vedolizumab has demonstrated therapeutic potential for EoE in two reported cases, the current data are limited to isolated case reports, corresponding to Level 4 evidence. Consequently, vedolizumab cannot be recommended for off-label use in the treatment of EoE at this time [89].

Interestingly, treatment with losartan, an angiotensin II receptor blocker, was associated with improvements in patient-reported outcome measures and the modulation of EoE Diagnostic Panel biomarkers; however, it did not lead to a consistent reduction in esophageal eosinophil counts. A subset of patients did exhibit histopathologic and endoscopic improvement, though no clear predictive factors for treatment response could be identified [90]. These findings suggest that while losartan may have a role in specific EoE phenotypes, possibly those with a dominant fibrostenotic component, its overall utility remains limited and requires further validation in larger, controlled clinical trials.

### 4.1. Small Molecules

The transcription factor FOXM1 is a key regulator of epithelial proliferation and inflammation in allergic asthma. To explore its role in epithelial disruption in eosinophilic esophagitis (EoE), recent studies have demonstrated that targeting FOXM1 restores epithelial homeostasis, reduces immune-mediated activity, and represents a potential novel treatment strategy for EoE [91]. Histologic success of maintenance therapy ≥ 48 weeks in RCTs was 86% [95% confidence interval, 71–96%] for corticosteroids and 79% (95% CI, 69–87%) for biologics. Dupilumab alone accounted for 82% (95% CI, 72–89%), whereas small molecules yielded 28% [92].

Through combined proteomic, transcriptomic, and functional analyses, researchers found increased IL-20 subfamily cytokine levels in active EoE, indicating their potential as therapeutic targets because of their involvement in suppressing barrier-protective genes like filaggrin [93].

### 4.2. Microbiome

Several studies report higher microbial load and the lack of variety, with a considerable overrepresentation of Haemophilus and reduced Firmicutes, such as Streptococcus and Lactobacillus, in EoE subjects [94,95,96]. Dysbiosis has been related to impaired barrier function and innate immune activation via Toll-like receptors (TLRs), perpetuating a Th2 inflammatory reaction in EoE. Emerging microbiome-targeted interventions enlighten the use of probiotics. *Lactococcus lactis* reduced IL-5 production and eosinophilic infiltration in a murine model, indicating that the animal models present encouraging results [97,98].

The adverse effects of probiotics and prebiotics have not yet been studied in EoE. Fiber intake can modulate intestinal microbiome profile. However, no data exists regarding the role of specific fiber fractions with a prebiotic mechanism (such as inulin-type fructans, fructo-oligosaccharides, and galactooligosaccharides) on the EoE. The association between high raffinose intake and EoE needs further investigation.

Fecal microbiota transplantation [FMT] could have potential for restoring esophageal microbiota. Modulating the esophageal microbiome represents an encouraging therapeutic frontier alongside current dietary, PPI, and steroid treatments. However, translating these insights to clinical practice requires human trials assessing probiotics, prebiotics, or FMT in EoE. Moreover, the potential risks, safety profile, and durability of such interventions have not been investigated in EoE.

Facchin et al. highlight that dysbiosis is a hallmark of EoE, and conventional current treatments already impact the microbiome. With preclinical evidence supporting probiotic and FMT strategies, there is a promising goal to develop microbiome-based therapies in EoE. However, clinical validation is the next critical step [99].

Research on the esophageal microbiome in EoE is still in its early stages, and current evidence remains largely preclinical.

Future studies are needed to clarify whether modulation of the esophageal or gut microbiome can meaningfully alter disease course and how these approaches might complement or interact with established treatments. Until well-designed clinical trials are conducted, microbiome-based therapies should be considered experimental and not ready for routine clinical application.

**Figure 2:**
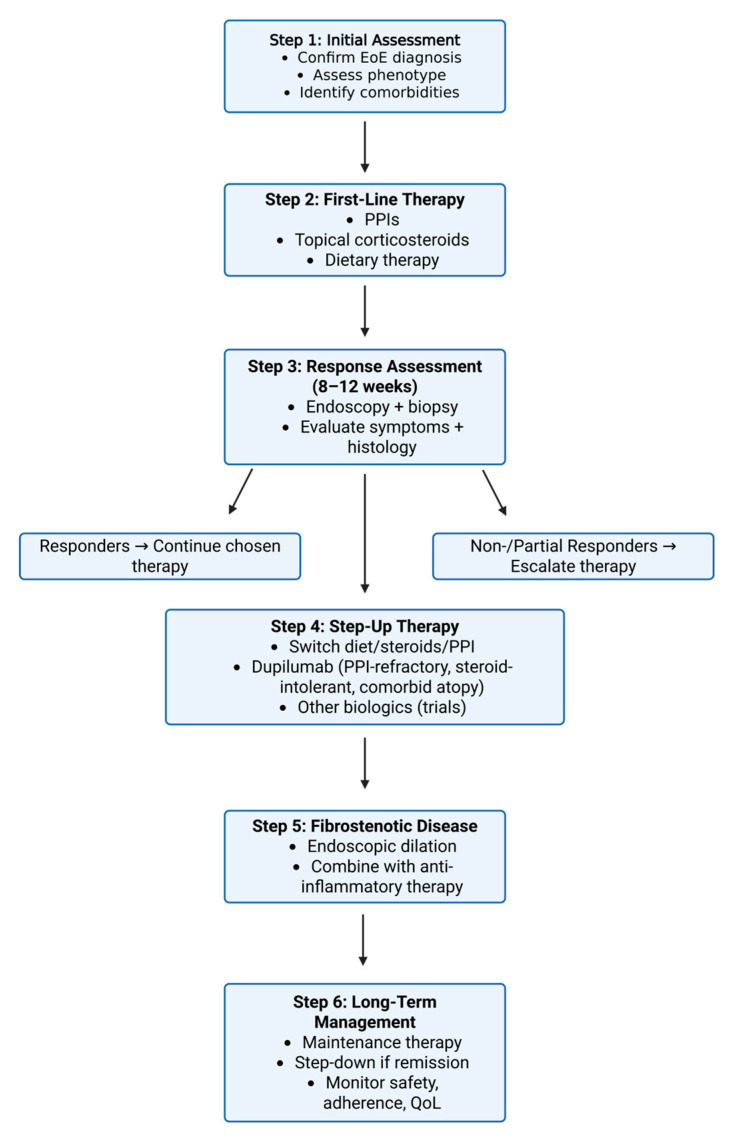
Proposed treatment algorithm for EoE (clinical framework).

According to current therapeutic options, we provide a treatment algorithm that balances advances (biologics, microbiome research) with challenges (adherence, fibrosis, costs) (Figure 2).

## 5. Conclusions

The therapeutic management of EoE remains challenging due to the complexity of the disease and the absence of a universally accepted standard of care. A major limitation is the lack of definitive curative therapy, necessitating long-term strategies aimed at symptom control and the prevention of complications.

The second issue is that, besides endoscopic dilation, there are no current pharmacologic treatments for improving the fibrostenotic phenotype. While resolving fibrosis and restoring the epithelium remain key treatment objectives, there is currently no drug capable of fully accomplishing either. A critical limitation of current evidence is the exclusion of fibrostenotic EoE patients from most clinical trials. This subgroup is characterized by long-standing disease, irreversible structural changes, and reduced responsiveness to anti-inflammatory therapies. Studying fibrostenotic EoE poses several challenges, including difficulties in defining appropriate endpoints beyond eosinophil counts, heterogeneity in disease progression, and ethical concerns regarding withholding established interventions such as dilation.

Future research should prioritize strategies for this population, including the development of antifibrotic agents, biomarkers to detect early tissue remodeling, and clinical trial designs that integrate histologic, endoscopic, and functional outcomes. Long-term prospective studies are also needed to determine whether early anti-inflammatory intervention can alter the natural course of disease and prevent fibrostenotic complications. Addressing this major gap will be essential for advancing precision medicine in EoE.

Another significant unmet need in EoE management is the lack of standardized therapeutic algorithms that incorporate ‘step-up’ and ‘step-down’ strategies tailored to disease severity and individual treatment response. Currently, it remains unclear whether long-term biological therapy can be safely spaced while maintaining remission or if concurrent treatments can be reduced and/or dietary restrictions relaxed. Additionally, it is unknown whether biological therapies can be temporarily discontinued or eventually withdrawn in patients who achieve sustained disease control.

In the last decade, several biological therapies, mainly used to treat severe eosinophilic asthma, have been evaluated in clinical trials for the management of EoE, and other newer agents are currently being assessed and investigated; hopefully, therapeutic choices will expand shortly.

EoE frequently coexists with other atopic conditions. These comorbidities not only contribute to the overall disease burden but may also influence treatment response. For example, patients with concomitant asthma or atopic dermatitis appear to benefit particularly from dupilumab, which targets shared Th2 pathways, providing dual therapeutic effects. Conversely, the presence of multiple atopic diseases may indicate a systemic atopic phenotype that could be less responsive to localized therapies, such as dietary elimination or topical corticosteroids.

Despite these observations, few clinical trials have reported subgroup analyses stratified by comorbid atopy [100,101]. Future research should investigate whether treatment responses differ in patients with single- versus multi-atopic disease and whether comorbidity profiles could help guide therapeutic selection. Such data would enrich precision medicine approaches and support more individualized treatment strategies in EoE.

Long-term safety profiles of emerging biologic agents remain incompletely understood, as most data are derived from short- to mid-term clinical trials.

Patient adherence is another critical issue, particularly with restrictive elimination diets or the long-term use of topical steroids, where sustained compliance is often low.

Furthermore, much of the evidence guiding current practice originates from controlled clinical trials, which may not reflect real-world patient populations that often include comorbidities, polypharmacy, and variable adherence. Real-world studies and registries are needed to better characterize treatment durability, safety, and patient-centered outcomes in diverse clinical settings. Addressing these challenges is essential for developing practical, sustainable, and patient-tailored management strategies.

Despite the availability of several effective therapies, important knowledge gaps remain regarding the optimal treatment approach, including the selection of first-line therapy, prediction of therapeutic response, assessment of disease severity and phenotypes, and prognostication of disease progression.

Future reviews and guidelines should focus on providing practical frameworks to guide therapy selection based on disease severity, treatment response, comorbidities, and patient preferences.

## Figures and Tables

**Table 1:** Efficiency of current treatment options on esophageal strictures, histologic remission, and symptom relief.

	Esophageal Strictures	Histological Remission	Symptom Relief
PPIs [29,30,31]	No	Plausible	Yes
Corticosteroids [36,37,38]	No	Plausible	Yes
Diet [25]	No	Plausible	Yes
Dupilumab [43,44]	No	Plausible	Yes
Dilation [12]	Yes	No	Yes

**Table 2:** Biologics investigated for EoE and their efficacy in the treatment of asthma.

Biologics	Approved for EoE	Approved for Asthma	Mechanism of Action
Dupilumab [73]	yes	yes	Anti-IL-4/ IL-13
Mepolizumab [74,77]	investigational	yes	Anti-IL-5
Reslizumab [75]	investigational	yes	Anti-IL-5
Cendakimab [76]	investigational	investigational	Anti-IL-13
Dectrekumab [57]	investigational	no data	Anti-IL-13
Benralizumab [78]	investigational	yes	Anti-IL-5
Lirentelimab [79]	investigational	investigational	Anti-Siglec-8
Etrasimod [66]	investigational	no data	Anti-S1P
Tezepelumab [79]	investigational	yes	Anti-TLSP

## Data Availability

Not applicable.
